# Histogenetic insights and genetic landscape of fibromatosis-like undifferentiated gastric carcinoma: a focused study

**DOI:** 10.1186/s12957-024-03479-2

**Published:** 2024-07-24

**Authors:** Yang-Kun Wang, Su-Nan Wang, Xing-Hai Liao, Zhi-Qiang Wang, Ping Li, Tian Yun, De-Qi Meng

**Affiliations:** 1 Department of Pathology, The Fourth People’s Hospital, 22 Longshan Industrial Zone, Nanwan Street, Longgang District, Shenzhen, 518123 China; 2 Shenzhen Polytechnic, Shenzhen, 518055 China; 3 Department of Pathology, Shenzhen Hospital, Southern Medical University, Shenzhen, 518101 China; 4 Department of Pathology, Foresea Life Insurance Guangzhou General Hospital, Guangzhou, 511300 China; 5 Department of Pathology, Peking University Shenzhen Hospital, Shenzhen, 518036 China; 6 Department of Pathology, The 989th Hospital of the Joint Logistics Support Force of the Chinese People’s Liberation Army, Luoyang, 471031 Henan China

**Keywords:** CTNNB1 gene, Fibromatosis-like undifferentiated carcinoma, Gastric cancer, HER2 gene, Histological features, Immunohistochemistry

## Abstract

**Background:**

The aim of this study was to elucidate the histogenesis and genetic underpinnings of fibromatosis-like undifferentiated gastric carcinoma (FLUGC), a rare pathological entity.

**Method:**

Through a detailed analysis of seven cases, including histopathological evaluation, CTNNB1 gene mutation screening, human epidermal growth factor receptor 2 (HER2) protein level quantification, and HER2 gene amplification assessment to identify the pathological and molecular characteristics of FLUGC.

**Results:**

Of the seven patients in this study, five were male and two were female (age: 39–73 years). Four patients presented with lesions in the gastric antrum and three had lesions in the lateral curvature of the stomach. Histopathologically, over 90% of the tumor consisted of aggressive fibromatosis-like tissue, including proliferating spindle fibroblasts and myofibroblasts and varying amounts of collagenous fibrous tissues. Undifferentiated cancer cells, accounting for less than 10%, were dispersed among the aggressive fibromatosis-like tissues. These cells were characterized by their small size and were relatively sparse without glandular ducts or nested mass-like structures. Immunophenotyping results showed positive expression of CKpan, CDX2, villin, and p53 in undifferentiated cancer cells; positive expression of vimentin in aggressive fibromatosis-like tissue; positive cytoplasmic expression of β-catenin; and focal cytoplasmic positive expression of smooth muscle actin (SMA). Genetic analysis did not reveal any mutations in the CTNNB1 gene test, nor was there amplification in the HER2 gene fluorescence in situ hybridization (FISH) test. Additionally, the Epstein-Barr encoding region (EBER) of in situ hybridization was negative; and the mismatch repair (MMR) protein was positive. Programmed cell death-1 (PD-1) was < 1–5%; programmed cell death ligand 1 (PD-L1): TPS = 1–4%, CPS = 3–8.

**Conclusion:**

The study highlights the significance of CTNNB1, HER2, EBER, and MMR as pivotal genetic markers in FLUGC, underscoring their relevance for diagnosis and clinical management. The rarity and distinct pathological features of FLUGC emphasize the importance of accurate diagnosis to prevent underdiagnosis or misdiagnosis and to raise awareness within the medical community.

## Background

Gastric cancer, the third leading cause of cancer mortality worldwide, demonstrates significant biological and genetic diversity, with multiple etiological factors that include both environmental and genetic factors. The disease is characterized by extensive morphological heterogeneity, including varied structures and growth patterns, degrees of cellular differentiation, and distinct histogenesis [1–3].

The commonly used methods of histopathological typing include the Borrmann classification, the World Health Organization (WHO) classification, and the Laurén classification [4–6]. In 1979, the WHO introduced an internationally standardized methodology of classification based on tissue origin and heterogeneity, where gastric cancer was classified into adenocarcinoma, adenosquamous carcinoma, squamous cell carcinoma, carcinoid, undifferentiated carcinoma, and unclassified carcinoma. Based on the histological features, subdivisions of adenocarcinomas included papillary adenocarcinoma, tubular adenocarcinoma, mucinous adenocarcinoma, and signet-ring cell carcinoma. Additionally, based on the degree of differentiation, they were classified as highly differentiated, moderately differentiated, and poorly differentiated adenocarcinomas. [7, 8].

In 1990, the WHO revised the histological classification of gastric cancer, where the new standard categorized gastric cancer into epithelial tumors and carcinoids. Epithelial tumors include adenocarcinomas (papillary adenocarcinoma, tubular adenocarcinoma, poorly differentiated adenocarcinoma, mucinous adenocarcinoma, and signet-ring cell carcinoma), squamous adenocarcinoma, undifferentiated carcinoma, and unclassified carcinoma. Gastrointestinal carcinoids refer to slow-growing neuroendocrine tumors with complex presentations. [9, 10].

The WHO diagnostic criteria put forth in 2010 classified gastric tumors as benign and malignant carcinomas, and these were distinguished based on factors such as the degree of differentiation, tumor size, infiltration depth, vascular invasion, and metastasis. Malignant carcinoid cells were identified by the presence of atypia that was above the moderate level, an increased nuclear fission index value (> 2/10 HPF), a tumor diameter > 1 cm, tumor invasion into the intestinal wall (intrinsic muscularis propria or extrinsic muscularis propria), or metastasis to the lymph nodes or the liver. Conversely, benign carcinoid cells exhibit less that moderate atypia, a nuclear fission index value of ≤ 2/10 HPF, and a tumor diameter of ≤ 1 cm without local infiltration or metastasis. [11, 12].

As per the 2019 WHO classification of tumors of the digestive system (5th edition), undifferentiated tumors were further categorized into large cell carcinomas with rhabdomyosiform phenotype, pleomorphic carcinoma, sarcomatoid carcinoma, and undifferentiated carcinoma with osteoblastic giant cells [13–15].

In summary, a diverse array of histomorphologies for undifferentiated gastric carcinoma has been identified in recent years. We have earlier reported the histomorphological features of three patients with fibromatosis-like undifferentiated gastric carcinoma [16], examined the tissue structure, proportional division of each tissue, and area calculation involving mixed gastric tumor [17], and examined the histopathological features and prognostic evaluation of gastric mucinous adenocarcinoma with signet-ring cells [18]. A thorough understanding of fibromatosis-like undifferentiated gastric carcinoma (FLUGC) is crucial for appreciating its clinical significance and potential therapeutic implications. FLUGC, characterized by aggressive fibromatosis-like tissue and undifferentiated cancer cells, poses considerable diagnostic and treatment challenges due to its rarity and unique histopathological features. Investigating the histogenesis, genetic mutations, and protein expressions associated with FLUGC can help to clarify the cellular mechanisms that are involved, improve diagnostic accuracy, and guide treatment decisions. Additionally, research into FLUGC contributes to the broader efforts to understand the heterogeneity of gastric cancer and identify therapeutic targets that may be applicable to other subtypes of the disease.

In this study, we analyzed seven cases of gastric fibromatosis-like structures with undifferentiated carcinoma to further examine the histogenesis, etiology, and associated genetic factors to deepen the understanding of the disease and offer clinicians a reliable pathological basis for precise treatment.

## Materials and methods

### Clinical data

For this study, data were collected from seven patients who underwent gastrectomy at the Fourth People’s Hospital of Longgang District, the Shenzhen Hospital of Southern Medical University, the Foresea Life Insurance Guangzhou General Hospital, and the Peking University Shenzhen Hospital, China, between September 2020 and May 2023. The histopathological diagnostic criteria used were based on the 2019 WHO classification of tumors of the digestive system [13] and the guidelines delineated in “*Gastric Tumor Pathology.*” [19] Among the seven patients, five were male and two were female (age: 39–73 years, 50.4 years on average). Four patients presented with lesions in the gastric antrum, while three had lesions in the lateral curvature of the stomach.

### Method

Within 30 min of surgical resection, the specimens were fixed for a duration of 8 to 48 h in freshly prepared 10% neutral buffered formalin (NBF), with a fixative-to-tissue volume ratio of 10:1. To ensure comprehensive sampling of tissue from the tumor region, tissues were selected based on criteria such as infiltration depth, varying colors, and differing textures. If the tumor diameter was < 3 cm, the whole tumor, including the peripheral area of the tumor, was sampled. In cases where the volume of the gastric tumor was ≥ 4 cm, 10 to 15 samples were collected, including at least four samples from the interface between the tumor and adjacent normal gastric tissue. The size of each sampled tissue was standardized at 2 cm × 1.5 cm × 0.3 cm. In addition, two samples were obtained from both the proximal and distal resection margins, and two samples were excised from the deepest infiltration point and the most adjacent plasma membrane. All lymph nodes and cancerous nodes were dissected and completely excised. The specimens were subjected to hematoxylin and eosin (HE) staining, microscopic observation, immunohistochemical analysis, and genetic testing.

### Immunohistochemistry

#### Routine immunohistochemical staining

The tissue section was deparaffinized using the EnVision two-step method, hydrated, and rinsed with distilled water. Then the section was placed in Tris-buffered saline (TBS) for 10 min. After blocking endogenous peroxidase for five minutes, the section was treated with TBS for 10 min. The section was incubated with each primary antibody (namely, CKpan, CK7, villin, CDX2, vimentin, SMA, S-100, desmin, CD99, ALK, CD34, CD68, CD163, GATA3, CD117, DOG1, p53, and ki-67) for 30 min at room temperature. After washing in TBS for 10 min, the section was incubated in EnVision™. After washing in TBS for 10 min, a secondary antibody was applied for 10 min. After incubating in the chromogenic substrate solution for 10 min, the section was rinsed with distilled water. Diaminobenzidine (DAB) was used for color development, and hematoxylin was used for re-staining. The known gastric mucosa sections were used as the positive control, and phosphate-buffered saline (PBS) buffer was used as the negative control instead of the primary antibody. The working solutions were purchased from Roche Diagnostics Ltd. (Shanghai), and the procedure was carried out in strict accordance with the manufacturer’s protocols provided in the kit.

#### Related gene protein testing

The EnVision two-step method was used to deparaffinize the tissue section, which was then hydrated and rinsed with distilled water. The section was placed in TBS for 10 min. After blocking endogenous peroxidase for five minutes, the section was processed with TBS for 10 min. The section was incubated with each primary antibody (MMR genes: MLH1, MSH2, PMS2, and MSH6; EGFR, β-catenin, and BRAF-V600E) for 30 min at room temperature. After washing in TBS for 10 min, the section was incubated in EnVision™. After washing in TBS for 10 min, the secondary antibody was applied for 10 min. After incubating in the chromogenic substrate solution for 10 min, the section was rinsed with distilled water. DAB was used for color development, and hematoxylin was used for re-staining. The known gastric mucosa sections were used as the positive control, and PBS buffer was used as the negative control instead of the primary antibody. The working solutions were purchased from Roche Diagnostics Ltd. (Shanghai), and the procedure was carried out as per the manufacturer’s instructions detailed in the kit.

## Epstein-Barr encoding region (EBER) in situ hybridization

All reagents, including proteinase K, the digoxin-labeled Epstein-Barr encoding region (EBER) probe, digoxin primary antibody, and the DAB display solution, were purchased from Roche Diagnostics Ltd. (Shanghai). Paraffin sections were baked in the drying oven at 65℃ for over 3 h and hydrated with xylene and gradient ethanol. They were then processed with proteinase K for 12 min and washed with PBS three times. Ten µl of the EBER probe was added dropwise, and the sections were incubated in a hybridization oven at 37℃ for 16 h and washed with PBS three times at 48℃. The primary antibody was added dropwise and incubated at 37℃ for one hour. Super Enhancer was added and allowed to react for 20 min, followed by the addition of an HRP-labeled polymer and placed for 30 min. DAB was used for color development, and re-staining were performed for one minute. After dehydration and clearing, the sections were sealed with neutral gum and observed under a microscope. A hybridization solution that did not contain the target base was taken as the negative control instead of the EBER probe.

## Programmed cell death-1 (PD-1)/ Programmed cell death ligand 1 (PD-L1)/ human epidermal growth factor receptor 2 (HER2) immunohistochemical testing

### Reagents and staining method

The immunohistochemical analysis in this study involved the assessment of expression levels of programmed cell death ligand 1 (PD-L1) (22C3), programmed cell death-1 (PD1) (2E5), and human epidermal growth factor receptor 2 (HER2) (SP3). The EnVision method was employed, and the experimental protocols adhered rigorously to the manufacturer’s guidelines. For the negative control, phosphate-buffered saline (PBS) was utilized in place of the primary antibody. Additionally, placental villi and lymph nodes served as positive controls for PD-L1 and PD1, respectively. The antibody ready-to-use kits and primary antibodies were purchased from Roche Diagnostics Ltd. (Shanghai).

### PD-1/PD-L1/HER2 result determination

The result was determined to be positive when the tumor mesenchymal lymphocyte PD-1 was localized at the cell membrane and/or the cytoplasm was brown. The tumor cell PD-L1 ratio score (TPS score) refers to the percentage of tumor cells with partial or complete membrane staining (≥ 1+) of all live tumor cells (negative and positive) in a sample. Additional requirements for the PD-L1 score were that there were at least 100 live infiltrating tumor cells and that the non-specific background staining intensity was less than 1+. The CPS score refers to the proportion of the total number of PD-L1-positive tumor cells, lymphocytes, and macrophages in all live tumor cells (negative and positive) in a sample.

Interpretation of HER2 positive results: the score was 0 if positivity was localized to the cell membrane and the membrane was not stained; a score of 1 + indicated that there was weak or faint membrane staining in cancer cells; a score of 2 + denoted that there was weak to moderate staining of the basement membrane, lateral membrane, or intact membrane of tumor cells; and a score of 3 + was given if there was strong staining of the basement membrane, lateral membrane, or intact membrane of the tumor cells.

## Fluorescence in situ hybridization (FISH) detection

### Reagents, probes, and fluorescence in situ hybridization (FISH) procedures

The paraffin pretreatment kit II (mainly consisting of a pretreatment solution and a protease solution) and the Path Vysion™ HER2 Probe Kit were purchased from Vysis. The pretreatment procedures of paraffin-embedded gastric cancer tissue sections and fluorescence in situ hybridization (FISH) procedures were performed in strict accordance with the literature [20] and protocols provided by the manufacturer in the kit.

### Determination of FISH results

The FISH gene testing of immunohistochemically stained positive sections involved initially identifying the HE-confirmed field of view under a 10× objective, followed by a detailed observation under a 40× objective. The result was considered satisfactory if the nuclei of cancer cells accounted for more than 75% of the hybridization signals. Subsequently, a 100× objective was used, and at least 30 isolated cancer cells with complete borders and no overlapping were counted.

The evaluation criteria for HER2 gene amplification were as follows: a positive result suggesting HER2 gene amplification was determined if the ratio of the HER2 gene copy number to the number of chromosome 17 in the nucleus of 30 cancer cells was > 2.2. Conversely, a negative result suggesting no HER2 gene amplification was concluded if the ratio was < 1.8. If the ratio ranged between 1.8 and 2.2, an additional region was selected for counting another 30 tumor cells to confirm the final result.

## CTNNB1 gene mutation detection

We used PCR Sanger sequencing technology to detect mutations in the CTNNB1 gene. Primers were designed to target specific exons of the CTNNB1 gene, particularly exon 3, as previous studies have shown that this region often undergoes mutations in certain tumors. After PCR amplification, direct sequencing was used to determine the gene sequence and analyze any possible gene variations.

For PCR and sequencing processes, reagent kits were purchased from Roche Diagnostics Co., Ltd. (Shanghai). All necessary components, including PCR buffer dNTPs, stable hot start DNA polymerase and DNA purification columns were provided in these test kits. The instructions provided by the manufacturer were strictly followed to ensure the repeatability of the experiment and the reliability of the results.

PCR amplification was performed on a PCR instrument with thermal cycling function with specific annealing temperatures and extension times optimized to ensure effective amplification of specific regions of the CTNNB1 gene. Post-amplification, PCR products underwent purification and were subsequently sent to a specialized sequencing service provider for Sanger sequencing. The sequencing outcomes were compared with known CTNNB1 gene sequences to detect any mutations or sequence variations.

## Results

### Clinical features and pathological staging

Among the seven patients, five were male and two were female (age: 39–73 years, 50.4 years on average). Four patients presented with lesions in the gastric antrum, while three had lesions in the lateral curvature of the stomach. Among the four patients who had Borrmann-type advanced gastric carcinoma, three had Type III (infiltrating ulcer type) and two had Type IV (diffuse infiltrating type). The pathological staging in two patients was pT4aN1Mx, while it was pT4aN2Mx in one patient.

### The occurrence and development process of FLUGC

**Fig. 1:**
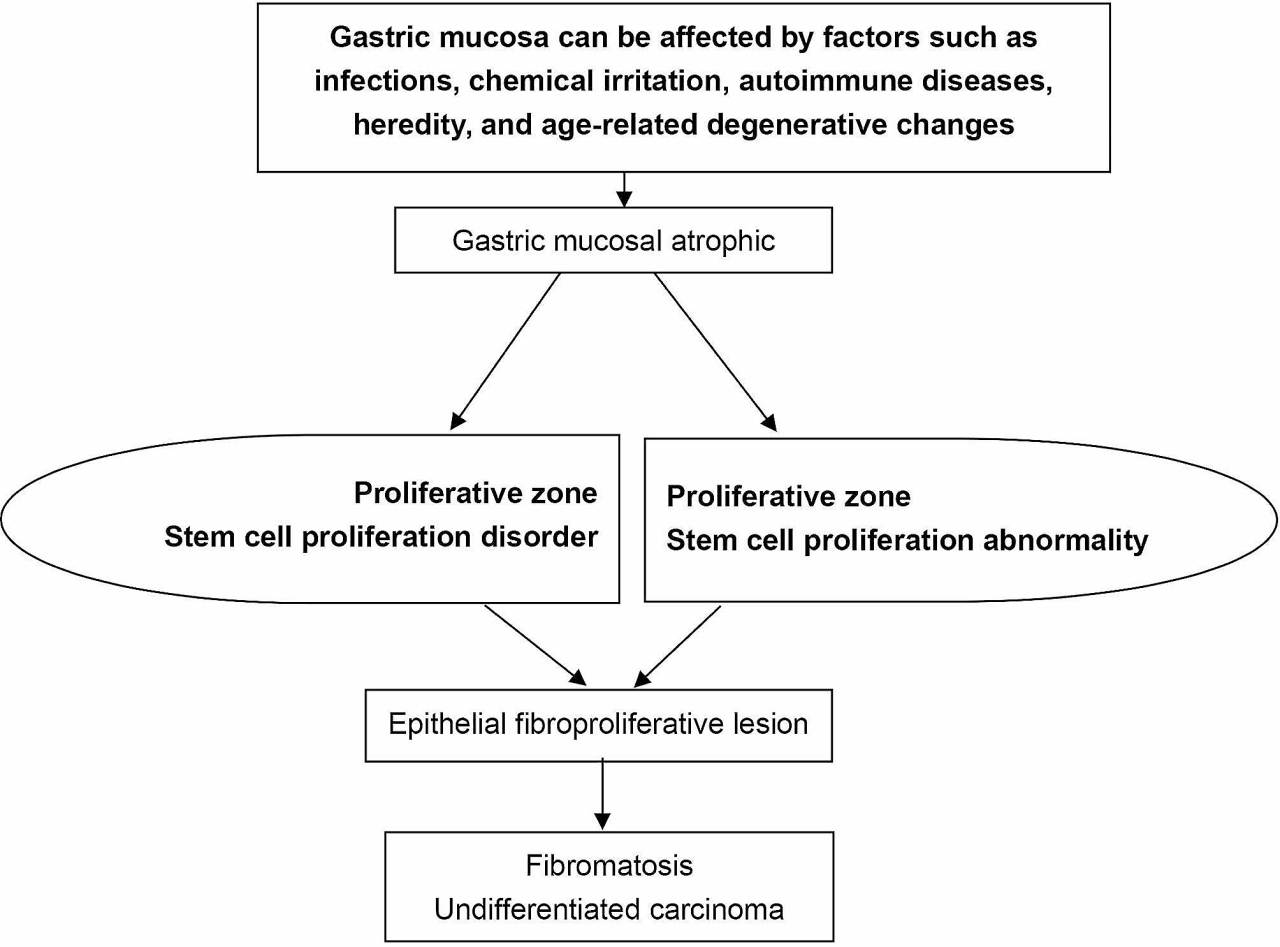
Schematic diagram of the occurrence and development of fibromatosis-like undifferentiated gastric carcinoma

Initially, there was an atrophic lesion of the gastric mucosa. Subsequently, there was disordered and abnormal proliferation in the proliferative zone, which caused an epithelial fibroproliferative lesion. This finally led to fibromatosis-like undifferentiated gastric carcinoma (Fig. 1).

### Histopathological features of FLUGC

**Fig. 2:**
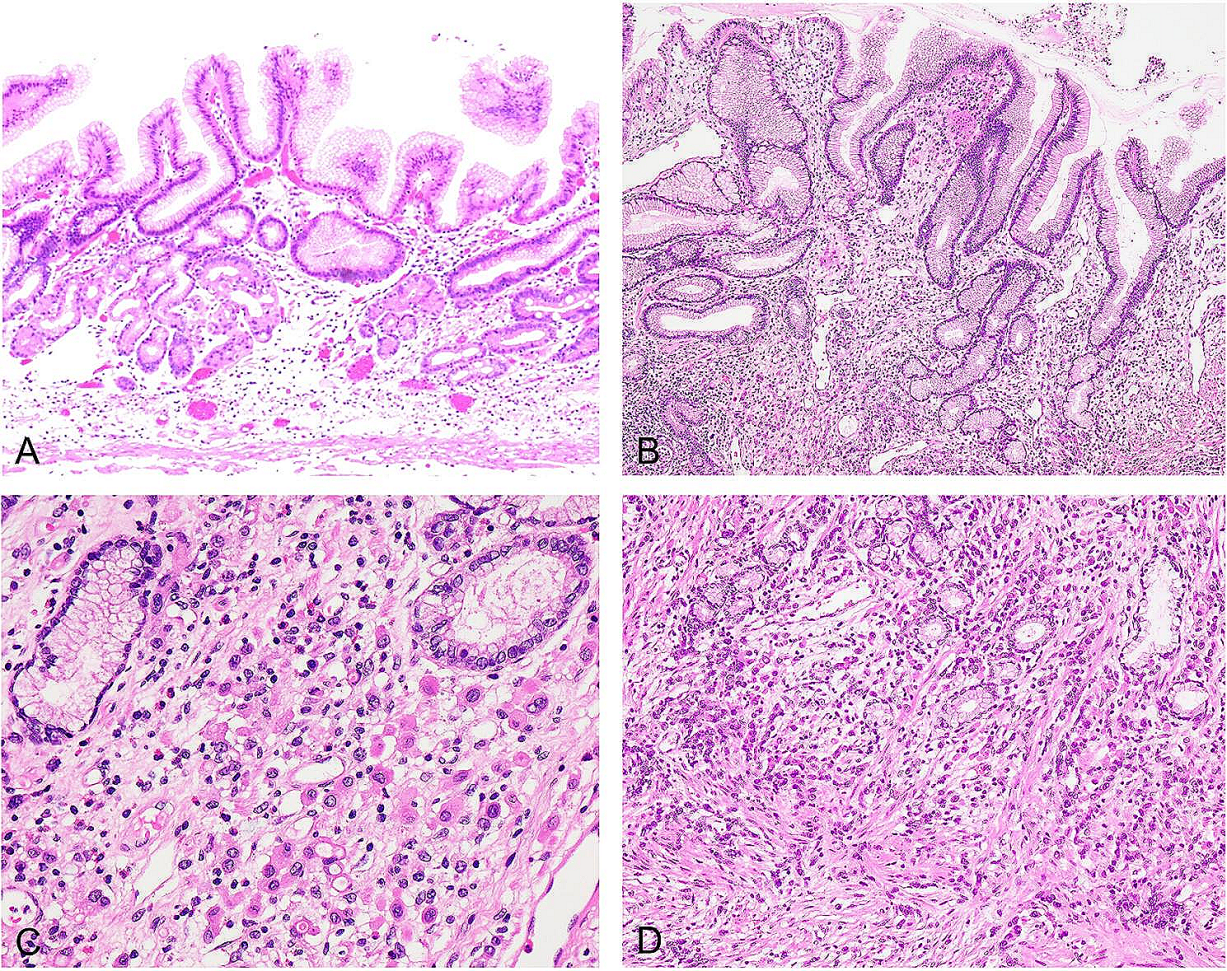
Formation of fibromatosis-like undifferentiated carcinoma of the stomach. **A**. Atrophic lesions of the gastric mucosa. Atrophy of the glands in the lamina propria results in a reduced number of gastric mucosal glands and thinning of the mucosa, H&E stain, ×200. **B**. Impaired proliferation in the proliferative zone. Ulcer-like structure with disrupted proliferation, destruction of the muscularis mucosa, H&E stain, ×200. **C**. The structure of abnormal proliferation. Disordered proliferation due to changes in the proliferation pattern and direction of proliferation of stem cells in the proliferative zone, H&E stain, ×400. **D**. Epithelial fibrous proliferative lesions. Persistently proliferating cells form sheet-like or nodular epithelial proliferative lesions. This disordered proliferation, combined with atrophy-induced fibrous tissue, creates neoplastic proliferative nodules composed of atypical epithelium and fibrous tissue, H&E stain, ×200

In all seven samples of fibromatosis-like undifferentiated gastric carcinoma, atrophic lesions of the gastric mucosa were observed in the gastric mucosa area (Fig. 2-A). Atrophy of the gastric mucosa led to impaired proliferation in the proliferative zone, which manifested in two distinctive ways: insufficient upward migration of the proliferative zone reduced the gastric foveal epithelium, while insufficient downward migration of the proliferative zone resulted in generalized atrophy of the gastric mucosal lamina propria glands (Fig. 2-B).

These changes in the proliferation pattern and direction of proliferation of stem cells in the proliferative zone lead to a state of disordered proliferation with the following morphological characteristics: cell atypia, disturbed mucosal structure, and abnormal differentiation (Fig. 2-C). Persistently proliferating cells formed block-shaped or nodular epithelioma proliferations. The disordered proliferation was intertwined with fibrous tissue resulting from atrophy, forming neoplastic proliferative nodules composed of heterogeneous epithelium and proliferative fibrous tissue—a condition termed the epithelial fibroproliferative lesion (Fig. 2-D).

**Fig. 3:**
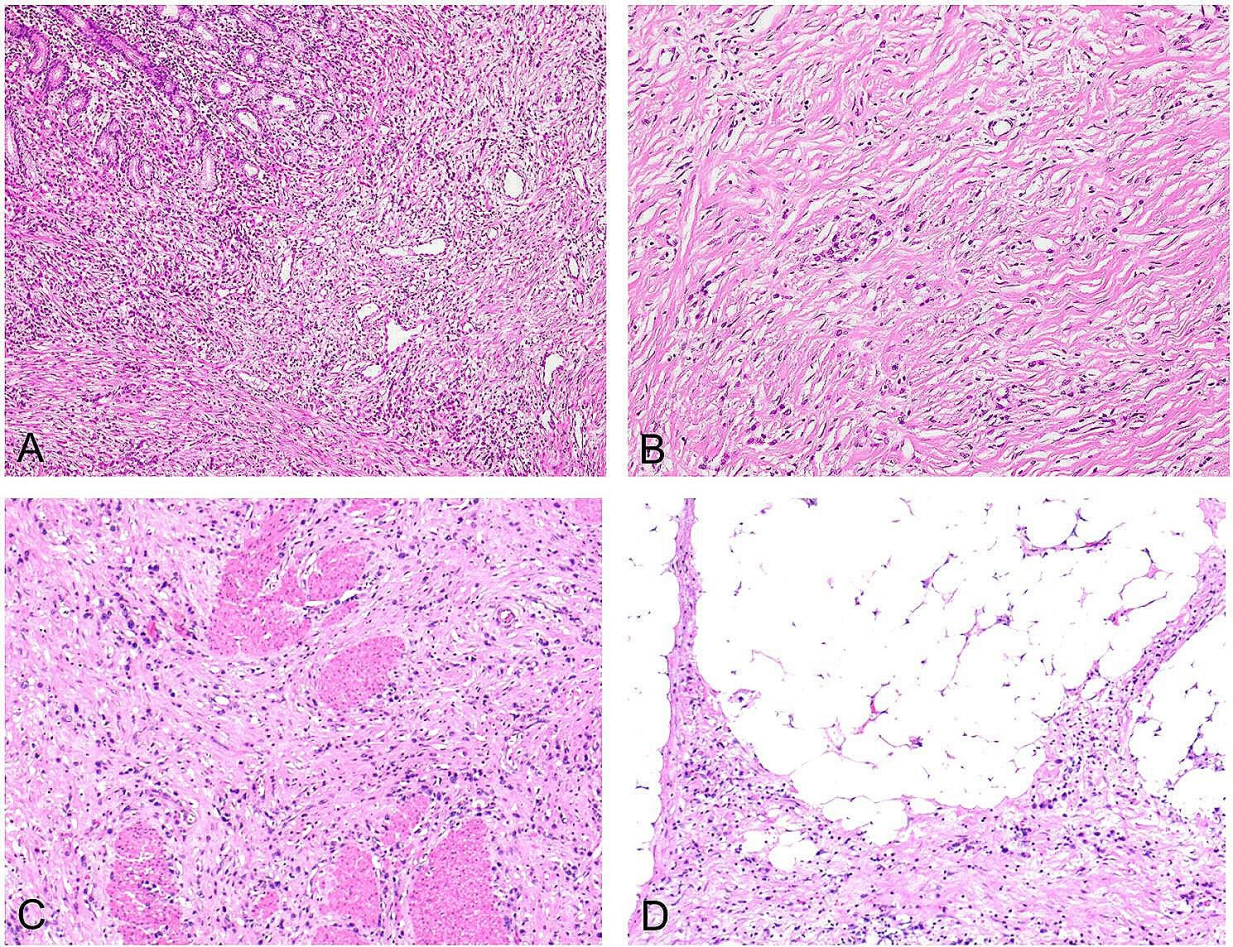
Histological characteristics of fibromatosis-like undifferentiated carcinoma of the stomach. **A**. Infiltrative growth in the mucosa and submucosa of the stomach. Mixed invasive fibromatosis-like tissue and undifferentiated carcinoma tissue, H&E, ×200. **B**. Histological composition. Undifferentiated carcinoma cells are scattered within the invasive fibromatosis-like tissue, distributed individually, and do not form glandular or nest-like structures. The cells are small in size and relatively sparse, accounting for less than 10%, H&E, ×200. **C**. When the tumor tissue invades the muscularis propria of the gastric wall, it fragments the smooth muscle tissue of the gastric wall into varying sizes of nest-like or disorganized clusters, H&E stain, ×200. **D**. Tumor tissue grows invasively along the interspaces of smooth muscle fibers, extending to the serosa, ×200

The epithelial fibroproliferative lesion in the mucosa continued to expand, break through the mucosal muscle, and invade into the submucosa, forming an unencapsulated invasive growth with aggressive fibromatosis tissue and undifferentiated cancerous tissue present (Fig. 3-A). Histologically, the aggressive fibromatosis-like tissue comprised the main components of the tumor cells, including proliferating spindle fibroblasts and myofibroblasts, and varying amounts of collagenous fibrous tissues, which accounted for more than 90% of the content.

Undifferentiated cancer cells were dispersed among the aggressive fibromatosis-like tissues and individually scattered without glandular ducts or nested mass-like structures. These cells were small in size and relatively sparse, accounting for about less than 10%. The growth pattern of the tumor was mainly characterized by aggressive fibromatosis; the spindle-shaped fibroblasts and collagen fibers were in bundles or interlaced weaves, and sometimes the collagen fibers formed striking broad and long bands. Tumor tissue invasion destroyed the mucosal muscle and progressed toward the submucosal layer, forming fibromatosis-like tissues (Fig. 3-B).

As the tumor tissue invaded the intrinsic muscular layer of the gastric wall, it fragmented the smooth muscle tissue of the muscular layer into nested clusters of varying sizes or disorganized laminated structures (Fig. 3-C). These structures were interspersed with smooth muscle fibers, and in some areas, the tumor tissue grew aggressively along the interstitial space of smooth muscle fibers all the way to the outer plasma membrane. The tumor tissue grew toward the space between adipose tissues outside the plasma membrane of the gastric wall in a crab-foot-like pattern (Fig. 3-D).

**Fig. 4:**
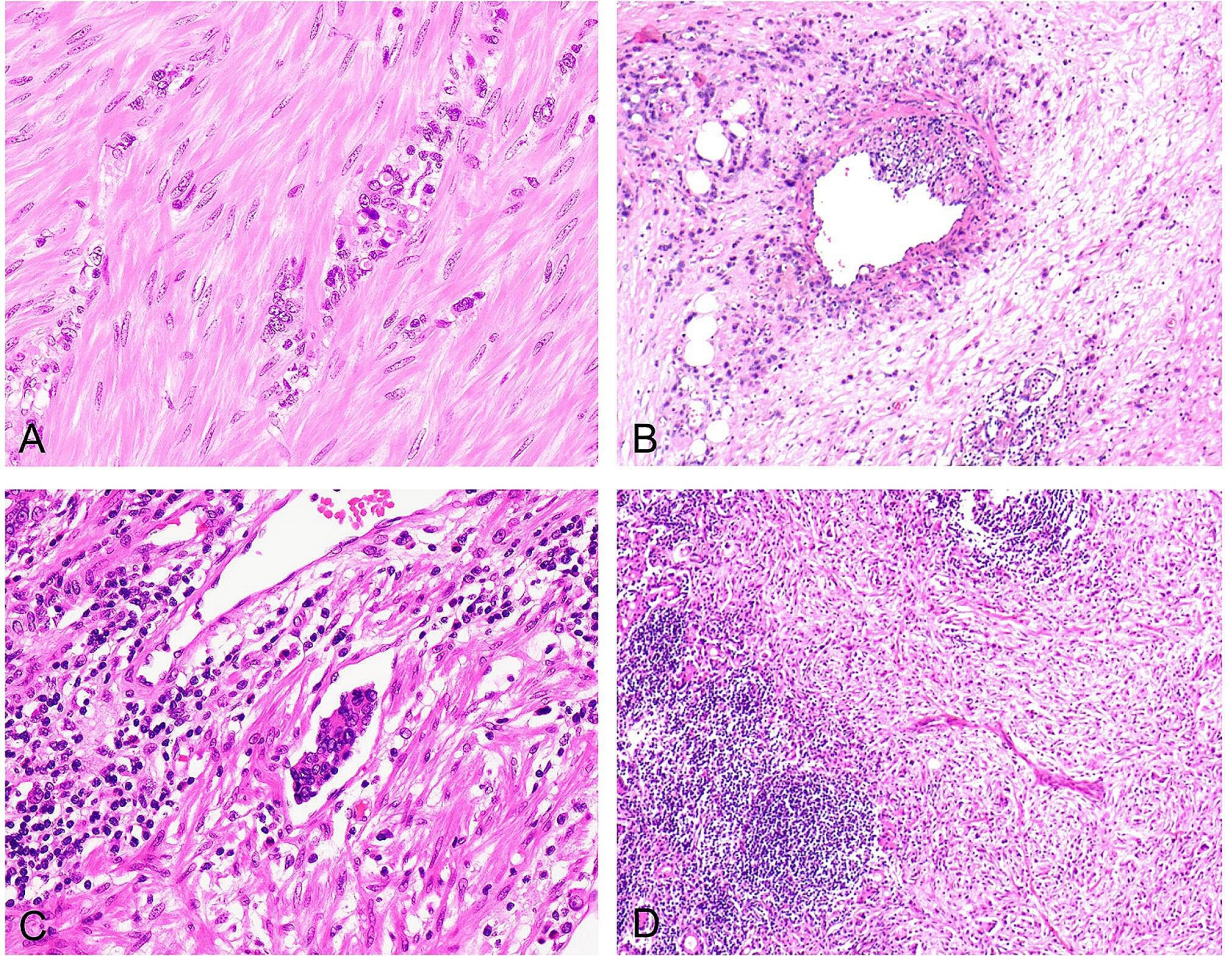
Characteristics of infiltration and metastasis in fibromatosis-like undifferentiated carcinoma of the stomach. **A**. Cytologically, the undifferentiated carcinoma cells are irregularly round or oval and of medium size. The nuclei are hyperchromatic, sometimes with finely granular chromatin, a prominent nucleolus, and occasionally eccentric nuclei, resembling plasmacytoid or signet-ring cell carcinoma. H&E, ×400. **B**. Carcinoma cells invading the vascular wall, H&E, ×200. **C**. Intravenous carcinoma thrombus, H&E stain, ×200. **D**. Lymph node metastasis showing the same histological structure as the primary lesion. There is significant fibrous connective tissue proliferation with carcinoma cells mostly scattered individually, not forming glandular or nest-like structures. H&E stain, ×100

Cytologically, the undifferentiated carcinomas were irregularly round or oval in shape and of medium size. The nuclei appeared deeply stained, with finely granular nuclear chromatin and a prominent nucleolus. Nuclear dislocation was occasionally observed, resembling plasma-like, signet-ring cancer cells, with nuclear mitosis ranging from 3 to 7% (Fig. 4-A). The morphological features of the directional invasiveness of tumor tissue capable of destroying blood vessels, lymphatic vessels, and neural tissues are crucial diagnostic features of fibromatosis-like undifferentiated gastric carcinoma. Cancer cells were observed invading the vessel wall (Fig. 4-B) or forming intravascular cancerous emboli (Fig. 4-C).

The tumor was infiltrated by a small number of lymphocytes, along with visible aggregates of regional lymphocytes. The fibromatosis-like undifferentiated gastric carcinoma was extremely aggressive, without any necrosis of tumor tissue. There was a high rate of lymph node metastasis, and all seven patients had lymph node metastasis. Lymph node metastases had the same histological structure as the primary lesion, characterized by extensive fibrous connective tissue hyperplasia and scattered cancer cells lacking glandular ducts or nested mass-like structures (Fig. 4-D).

### Immunohistochemical results

#### Results of routine immunohistochemical staining

**Fig. 5:**
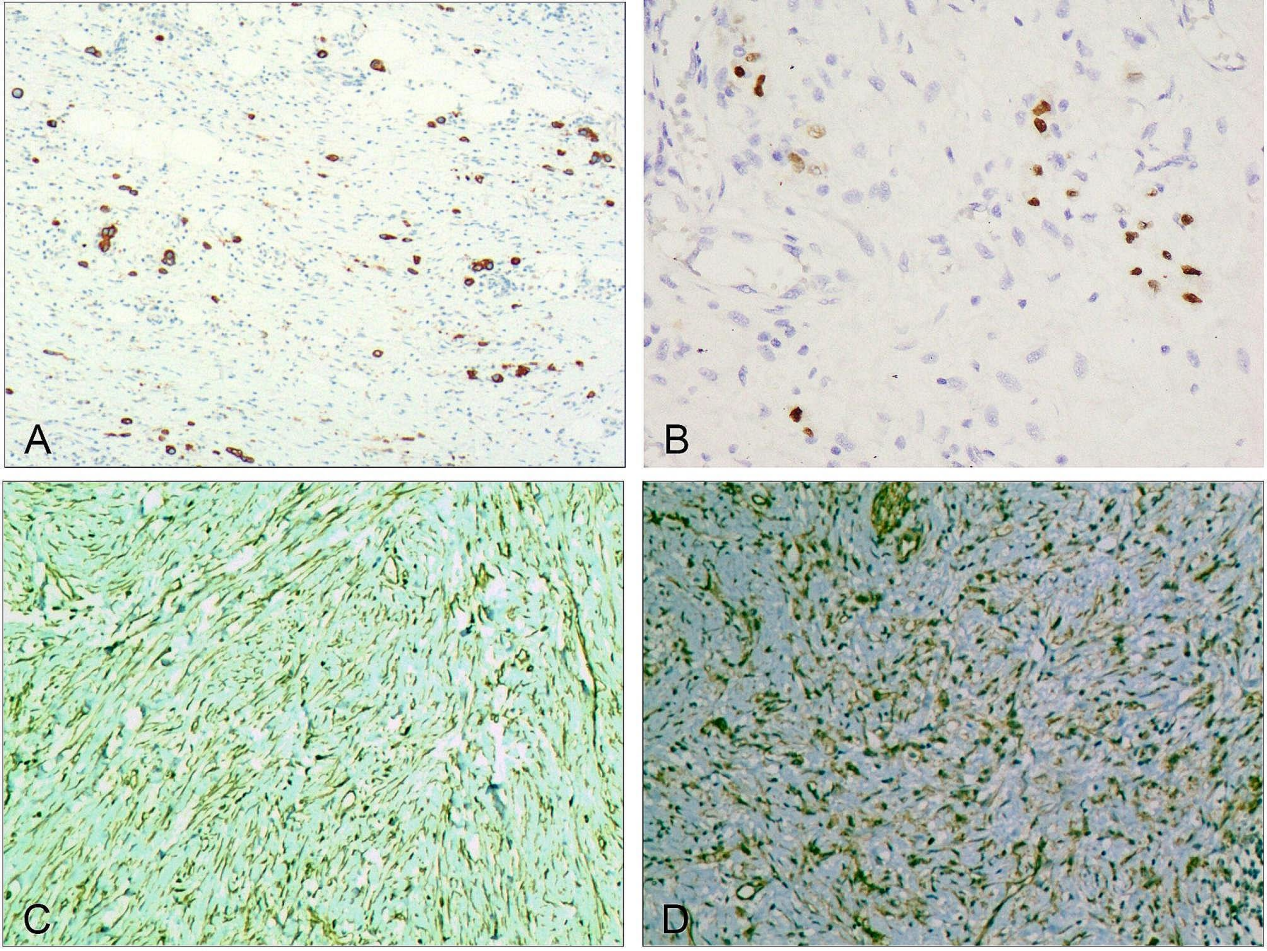
Immunohistochemical characteristics of fibromatosis-like undifferentiated carcinoma of the stomach. **A**. Cytoplasmic positive expression of CKpan in undifferentiated carcinoma cells. EnVision method, ×200. **B**. Nuclear positive expression of CDX2 in undifferentiated carcinoma cells. EnVision method, ×400. **C**. Positive expression of vimentin in the cytoplasm and nucleus of fibromatosis-like tissue. EnVision method, ×200. **D**. Cytoplasmic positive expression of β-catenin in fibromatosis-like tissue. EnVision method, ×200

**Fig. 6:**
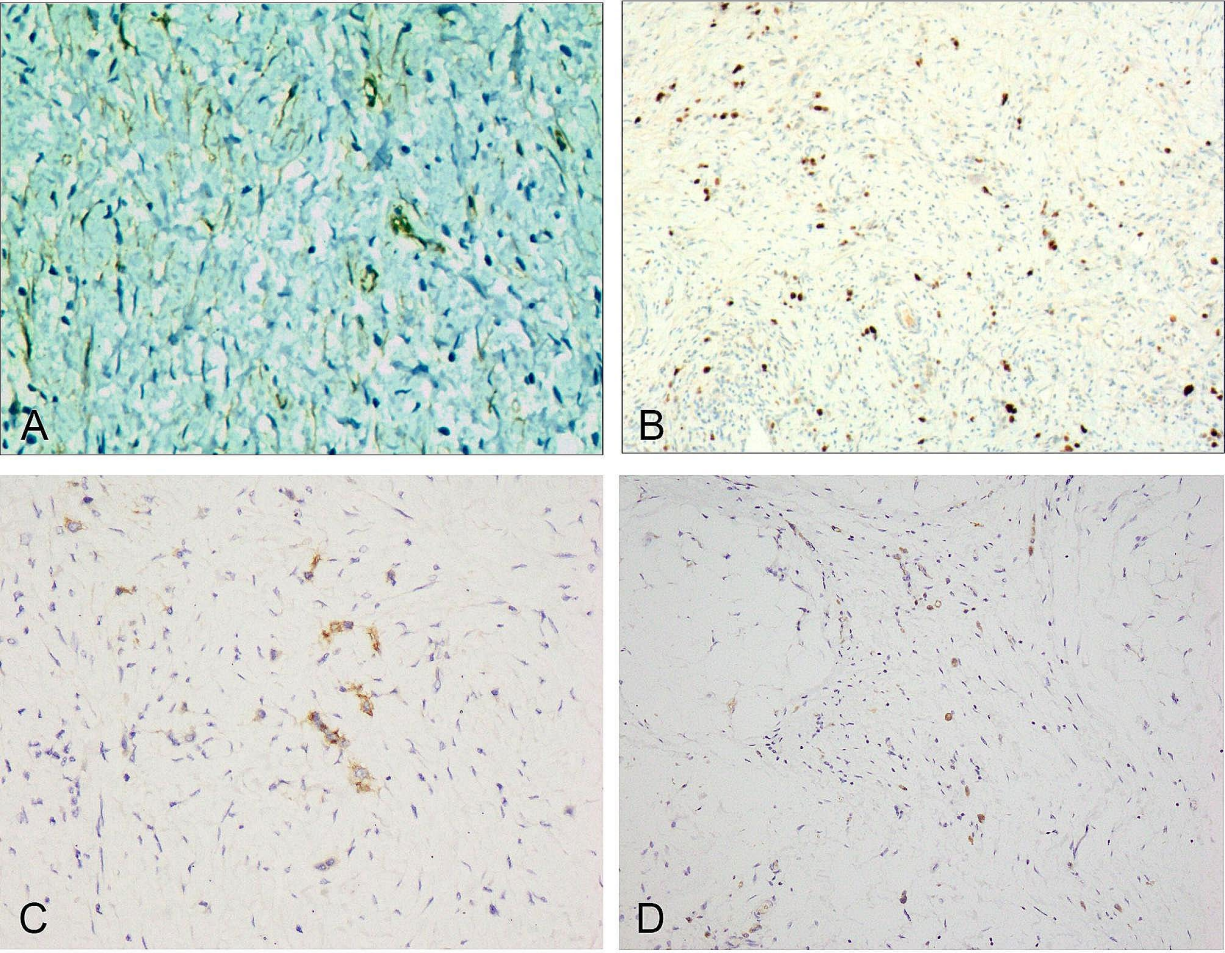
Immunohistochemical characteristics of fibromatosis-like undifferentiated carcinoma of the stomach. **A**. Focal cytoplasmic positive expression of SMA in fibromatosis-like tissue. EnVision method, ×200. **B**. Nuclear positive expression of Ki-67 in 70–80% of undifferentiated carcinoma cells. EnVision method, ×400. **C**. Cytoplasmic positive expression of EBER in undifferentiated carcinoma cells. EnVision method, ×200. **D**. Cytoplasmic positive expression of BRAF-V600E in undifferentiated carcinoma cells. EnVision method, ×200

Undifferentiated cancer cells showed positive expression of CKpan (Fig. 5-A), CK20, villin, CDX2 (Fig. 5-B), and p53. Spindle fibroblasts, myofibroblasts, and varying amounts of collagen fibers showed strong positive expression of vimentin (Fig. 5-C), positive cytoplasmic expression of β-catenin (Fig. 5-D), and focal cytoplasmic positive expression of SMA (Fig. 6-A). The expression of S-100, desmin, CD99, ALK, CD34, CD68, CD163, GATA3, CD117, and DOG1 were all negative. Ki-67-positive cells accounted for 70–80% of the proliferating cells (Fig. 6-B).

#### Results of related gene tests

MMR genes: MLH1, MSH2, PMS2, and MSH6 were positive. Four cases showed positive expression of EGFR (Fig. 6-C), and five cases showed positive expression of BRAF-V600E (Fig. 6-D), as shown in Table 1.

**Table 1:** Results of genetic tests related to fibromatosis-like undifferentiated gastric carcinoma

Case	Age	Gender	HER2 protein expression	HER2 gene amplification	CTNNB1 gene mutation test	EGFR	EBER in situ hybridization	BRAF-V600E	PD-1	PD-L1	MLH1, MSH2, PMS2 and MSH6
1✓	73	F	1**+**	-	-	+	-	+	2%	TPS = 1%; CPS = 3	+, +, +, +
2	68	M	-	-	-	-	-	+	< 1%	TPS = 3%; CPS = 4	+, +, +, +
3	55	M	1**+**	-	-	+	-	-	5%	TPS = 2%; CPS = 8	+, +, +, +
4	39	F	-	-	-	+	-	+	2%	TPS = 2%; CPS = 5	+, +, +, +
5	53	M	-	-	-	-	-	-	< 1%	TPS = 3%; CPS = 4	+, +, +, +
6	57	M	1**+**	-	-	+	-	-	3%	TPS = 1%; CPS = 6	+, +, +, +
7	48	M	-	-	-	-	-	+	2%	TPS = 4%; CPS = 7	+, +, +, +

### EBER in situ hybridization

All seven cases were negative.

### HER2 gene amplification and protein expression rate

HER2 protein-positive expression was localized to the cell membrane, and three cases had a score of 1+. The HER2 gene FISH test showed no hybridization.

### PD1/PD-L1 expression results

PD-L1-positive cells in gastric cancer tissues showed multifocal and patchy expression; PD-1-positive expression in tumor mesenchymal lymphocytes was characterized by scattered or patchy aggregates, often forming focally distributed lymphocyte aggregates. PD-1 expression level was < 1–5%; PD-L1: TPS range was 1–4%, CPS range was 3–8, as shown in Table 1.

### CTNNB1 gene mutation test result: exon

**Fig. 7:**
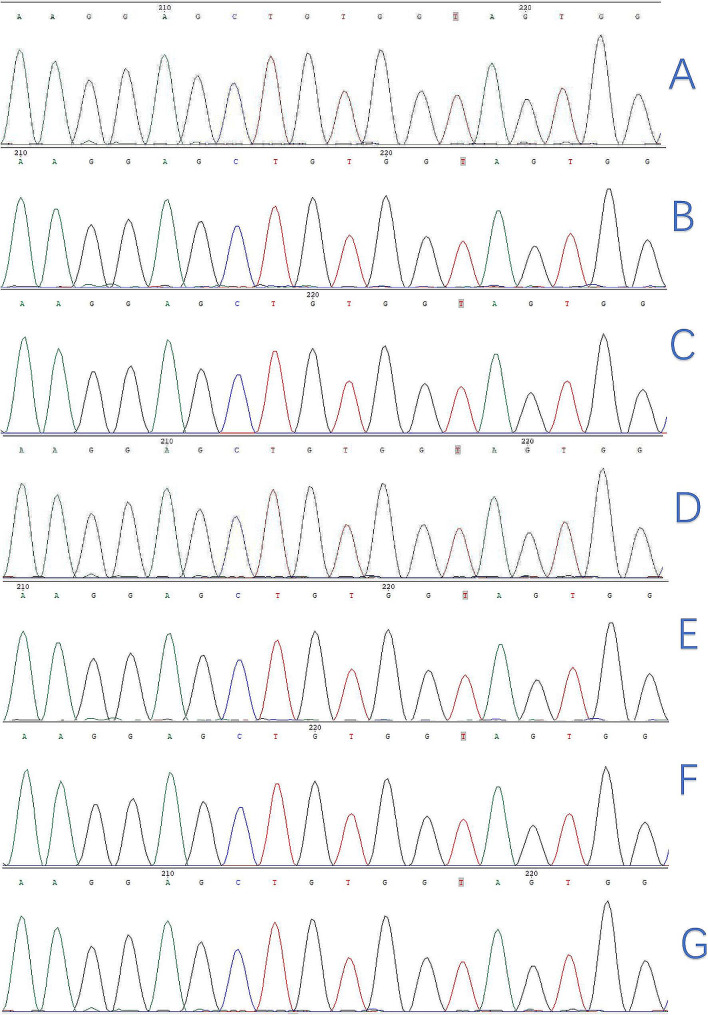
Exon3 sequencing results of the CTNNB1 gene

Exon3. Mutation type: point mutation. Test result: no mutation was detected. The CTNNB1 gene Exon3 sequencing result is shown in Fig. 7.

### Follow-up

The follow-up was conducted until May 31, 2023, spanning a period of 32 months, from September 2020 to May 2023. Follow-up communication was in the form of calls or letters to the patients or their families.

## Discussion

The WHO has classified *Helicobacter pylori* (*H. pylori*) infection as a carcinogen for gastric cancer, and chronic gastritis, chronic atrophy, and intestinal epithelialization have all been linked to *H. pylori* infection. Preventing gastric cancer by detecting and treating *H. pylori* has become increasingly common. [21, 22] In our previous study, we found that *H. pylori* infection disrupted stem cell proliferation in the proliferative zone. This disruption manifested in two ways: excessive upward migration of the proliferative zone formed generalized segmental papillomatous hyperplasia, while insufficient downward migration of the proliferative zone resulted in generalized segmental atrophy of the gastric mucosal lamina propria glands. Finally, these changes caused generalized segmental papillary hyperplasia and the formation of laminar gland atrophy [23].

In the context of the current study, we hypothesized that *H. pylori* infection-induced gastric mucosal atrophy contributes to the development of fibromatosis-like undifferentiated gastric carcinoma. We observed that atrophic lesions in the gastric mucosa could lead to proliferative dysfunction in the proliferative zone. The proliferation pattern or direction of migration of stem cells from the top of the gastric fundus gland to the deeper regions of the foveola gastrica was altered. This disturbance disrupted the dynamic equilibrium necessary for normal tissue homeostasis, thus forming a state of disordered proliferation and subsequent abnormal tissue proliferation.

Three key morphological features in fibromatosis-like undifferentiated gastric carcinoma were identified in this study: cellular atypia, disturbed mucosal structure, and abnormal differentiation. Persistently proliferating cells formed block-shaped or nodular epithelioma proliferations. This disordered proliferation was intertwined with fibrous tissue resulting from atrophy, forming neoplastic proliferative nodules composed of heterogeneous epithelium and fibrous tissue, known as epithelial fibroproliferative lesions. Proliferative anomalous nodules in the mucosa gradually enlarged, grew, and infiltrated into the submucosa, forming neoplastic lesions.

Histologically, the main components of the tumor included proliferating spindle fibroblasts and myofibroblasts, as well as varying amounts of collagenous fibrous tissues, comprising more than 90% of the content. Undifferentiated cancer cells were dispersed among the invasive fibromatosis-like tissues; these cells were small in volume and relatively sparse without glandular ducts or nested mass-like structures, accounting for less than 10%. This development of fibromatosis-like undifferentiated gastric carcinoma was attributed to gastric mucosal atrophy. In this study, we proposed five stages in the progression of genetic characteristics based on changes in tissue development.

FLUGC is characterized by a background resembling fibromatosis, with undifferentiated cancer cells dispersed in the tissue. Histologically, it is similar to fibromatosis, exhibiting strong invasiveness without tissue necrosis. Undifferentiated gastric carcinoma was found to vary widely in terms of histological patterns and prognostic outcomes. [24, 25].

We identified five essential criteria for a histopathological diagnosis of FLUGC: (1) The tumor lacks clear borders and is characterized by strong invasion and destruction of surrounding tissues without causing tissue necrosis. (2) The aggressive fibromatosis-like tissue comprises the main tumor components, including proliferating spindle fibroblasts and myofibroblasts and varying amounts of collagenous fibrous tissues, which account for more than 90% of the content; undifferentiated cancer cells are dispersed among the aggressive fibromatosis-like tissues; the cells are small in volume and relatively sparse, without glandular ducts or nested mass-like structures, accounting for less than 10%. (3) The tumor tissue demonstrates directional invasiveness, destroying blood vessels, lymphatic tubes, and neural tissues. (4) Cytologically, undifferentiated carcinoma could be irregularly round or oval and 5–6 times the size of lymphocytes. The nucleus appears deeply stained, with finely granular nuclear chromatin and a prominent nucleolus, with occasional nucleus dislocation, resembling plasma-like or signet-ring cancer cells. (5) Lymph node metastases disrupt the lymph node capsule, and they mirror the histological structure of the primary lesion. (6) With respect to immunophenotyping, positive expression of CKpan, CDX2, villin, and p53 in undifferentiated cancer cells; positive expression of vimentin in aggressive fibromatosis-like tissue; positive cytoplasmic expression of β-catenin; and focal cytoplasmic positive expression of SMA can be found. (7) Related genes: there are no mutations in the CTNNB1 gene test, no amplification in the HER2 gene FISH test, a negative EBER of in situ hybridization, and a positive MMR protein.

Aggressive fibromatosis refers to the abnormal proliferation of clonal fibroblasts and myofibroblasts in deep soft tissues, characterized by infiltrative growth into the surrounding soft tissues and a high tendency for local recurrence [26]. In a study on 101 patients with aggressive fibromatosis, there were 17 recurrences after 41 months, with a cumulative 5-year recurrence rate of 22.8%, and the CTNNB1 mutation was found in 76 patients [27]. In this study, we found that proliferating spindle fibroblasts and myofibroblasts and varying amounts of collagenous fibrous tissues in fibromatosis-like undifferentiated carcinoma accounted for over 90% of the tumor content, and the CTNNB1 mutation was not detected in any of the seven patients.

The HER2 gene amplification state is an important marker for evaluating treatment options for gastric cancer. FISH is the gold standard for detecting the status of the HER2 gene, and HER2 expression or HER2 amplification can be used to test the effectiveness of trastuzumab [28]. In an earlier study, we studied differentiated gastric adenocarcinoma and found a HER2 protein expression rate of 40.8%, of which HER2 protein 3 + accounted for 10.8%, HER2 protein 2 + accounted for 14.2%, and HER2 protein 1 + accounted for 15.8%, while the HER2 gene amplification rate was 38.8% [20]. In our current study on FLUGC, none of the patients showed undifferentiated cancer cell HER2 protein 3 + expression, and HER2 gene amplification was absent.

The Epstein-Barr virus (EBV) is closely related to the development of gastric cancer, and EBER in situ hybridization has become a commonly used test for EBV. EBV infection was found to occur in the early stage of gastric cancer, and tumor cells infected with EBV infection could develop monoclonal proliferation, thus leading to EBER expression in almost all cancer cells in EBV-positive gastric cancer tissues. [29, 30] However, in our study, the FLUGC cases examined showed negative expression.

The immune checkpoint inhibitor PD-1/PD-L1 is recognized for its involvement in cancer immune evasion. Elevated expression of the immune checkpoint inhibitor genes PD-1/PD-L1 has been observed in patients diagnosed with gastritis, gastric ulcers, and gastric cancer [31]. In our study, the expression of PD-1/PD-L1 in FLUGC was not prominent (PD-1 < 1–5%; PD-L1: TPS = 1–4%, CPS = 3–8), indicating the inactive immune response of fibromatosis-like undifferentiated gastric carcinoma.

## Conclusion

In this study, we postulated that *H. pylori* infection may induce atrophic lesions in the gastric mucosa, potentially serving as the origin of FLUGC. We observed that FLUGC is primarily characterized by aggressive fibromatosis-like tissue, comprising over 90% of the tumor mass. Within this tissue, undifferentiated cancer cells are sparsely dispersed, lacking glandular ducts or nested mass-like structures, and accounting for less than 10% of the tumor volume.

FLUGC exhibits distinctive features in its histogenesis, morphology, biological behavior, immunophenotype, and genetic profile, distinguishing it from other gastric cancer subtypes. It is imperative to meticulously identify FLUGC to avoid underdiagnosis and misdiagnosis. Enhancing awareness of FLUGC among clinicians and pathologists can facilitate early and appropriate management strategies, potentially improving patient outcomes.

## Data Availability

No datasets were generated or analysed during the current study.

## Abbreviations

WHO: World Health Organization
AF: Aggressive fibromatosis
HER2: Human epidermal growth factor receptor 2
FISH: Fluorescence in situ hybridization
PD-L1: Programmed cell death ligand 1
PD-1: Programmed cell death − 1
